# Research status and future prospects of extracellular vesicles in primary Sjögren’s syndrome

**DOI:** 10.1186/s13287-022-02912-1

**Published:** 2022-06-03

**Authors:** Jingwen Zhao, Qi An, Xueqing Zhu, Baoqi Yang, Xinnan Gao, Yuhu Niu, Liyun Zhang, Ke Xu, Dan Ma

**Affiliations:** 1 Third Hospital of Shanxi Medical University, Shanxi Bethune Hospital, Shanxi Academy of Medical Sciences, Tongji Shanxi Hospital, Taiyuan, 030032 China; 2 Department of Biochemistry and Molecular Biology, Shanxi Medical University, 56, Xinjian South Rd., Taiyuan, China

**Keywords:** Primary Sjögren’s syndrome, Extracellular vesicles, Exosomes, Mesenchymal stem cells

## Abstract

Primary Sjögren’s syndrome (pSS) is a diffuse connective tissue disease characterized by the invasion of exocrine glands such as lacrimal and salivary glands, abnormal proliferation of T and B lymphocytes, and infiltration of tissue lymphocytes. With the development of modern medicine, although research on the pathogenesis, diagnosis, and treatment of pSS has made significant progress, its pathogenesis has not been fully understood. Meanwhile, in the era of individualized treatment, it remains essential to further explore early diagnosis and treatment methods. Exosomes, small vesicles containing proteins and nucleic acids, are a subtype of extracellular vesicles secreted by various cells and present in various body fluids. Exosomes contribute to a variety of biological functions, including intercellular signal transduction and pathophysiological processes, and may play a role in immune tolerance. Therefore, exosomes are key to understanding the pathogenesis of diseases. Exosomes can also be used as a therapeutic tool for pSS because of their biodegradability, low immunogenicity and toxicity, and the ability to bypass the blood–brain barrier, implying the prospect of a broad application in the context of pSS. Here, we systematically review the isolation, identification, tracing, and mode of action of extracellular vesicles, especially exosomes, as well as the research progress in the pathogenesis, diagnosis, and treatment of pSS.

## Introduction

Primary Sjögren’s syndrome (pSS) is a chronic autoimmune disease characterized by lymphocyte proliferation and progressive damage to exocrine glands. The etiology of pSS is unclear and is presumed to be related to genetic, environmental, and immune factors, with a prevalence rate of approximately 0.5–5%. In China, with a prevalence rate of approximately 0.3–0.7%, pSS is the most common autoimmune connective tissue disease, primarily affecting middle-aged women, with complex and diverse clinical manifestations, resulting in dry surfaces of the major mucous membranes, such as the mouth, eyes, nose, throat, and vagina. Approximately 30% of patients develop multi-system lesions involving the lungs, kidneys, and nervous system, and an estimated 5% of patients develop life-threatening malignant lymphoma [1, 2]. This disease is divided into two categories, primary and secondary SS. Current research on the pathogenesis and diagnostic treatment of pSS lags behind that of other autoimmune diseases as it is dominated by symptomatic treatment, lack of effective therapies, and poor patient outcomes, prompting continuous exploration of its pathogenesis and the search for effective treatment strategies.

Extracellular vesicles (EVs) are cell-derived membranous structures released by various cells. The exosome is a subtype of EV with a 50–150 nm diameter. Studies have shown that exosomes are associated with immune response, viral pathogenicity, pregnancy, cardiovascular diseases, central nervous system-related diseases, and cancer progression. Exosomes reportedly exist in all biological body fluids and can be easily obtained by collecting biological body fluid samples. Exosome-based biological humoral examinations highlight their potential value in the diagnosis and prognosis of patients with pSS, cancer, and other diseases. Exosomes have played a potential role in the diagnosis and treatment of many diseases. Although non-steroidal anti-inflammatory drugs (NSAIDs), glucocorticoids, immunosuppressants, and specific biological agents such as CD20 monoclonal antibodies, are effective in treating pSS, these drugs pose side effects and cannot repair damaged tissue. Exosomes can transport different effective therapeutic carriers, including short interfering RNAs, antisense oligodeoxynucleotides, chemotherapeutic drugs, and immunomodulators, efficiently delivering them to the desired targets. The lipid and protein composition of exosomes can affect their pharmacokinetic profiles, and their natural components may play a role in improving bioavailability and reducing adverse reactions. An accurate understanding of methods such as isolation, purification, and identification of exosomes is essential to studying the mechanisms and potential roles of exosomes; thus, we first describe the progress in exosomes isolation, purification, identification, and tracing, followed by a review of their applications in the pathogenesis, diagnosis, and treatment of pSS.

## Overview of extracellular vesicles

**Fig. 1:**
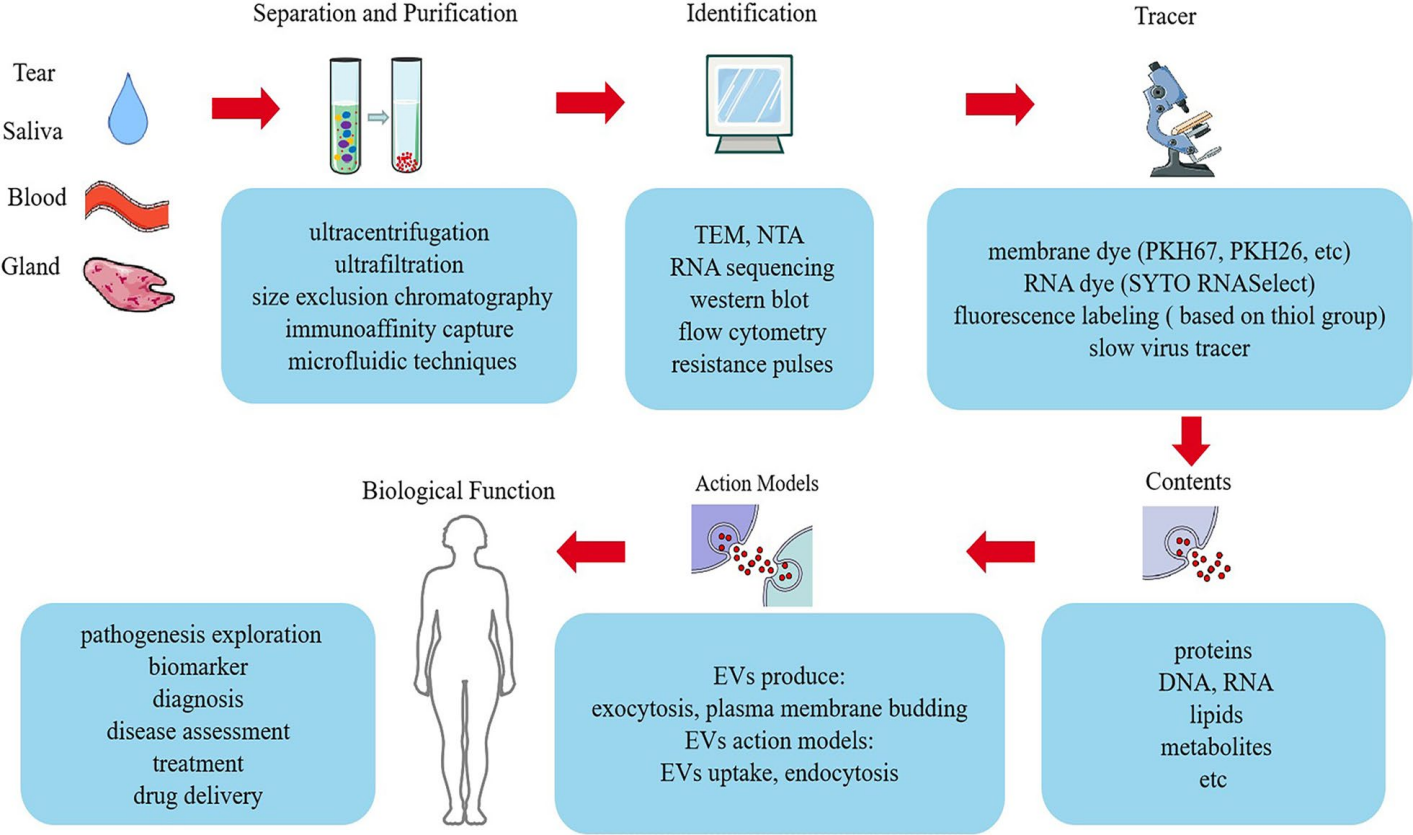
Separation, purification, identification, tracing, contents, mode of action, and biological functions of EVs

Although the presence of vesicles within mammalian tissues and body fluids was first described in the 1960s, it was not until recently that the technical term “extracellular vesicles” (EVs) was proposed to define various cell-derived membranous structures. EVs can be divided into three types according to their diameter: exosomes, microvesicles, and apoptotic bodies [3, 4]. Exosomes are vesicles 50–150 nm in diameter originating from endosomes. The continuous invagination of the plasma membrane eventually forms polycysts, which can intersect with other vesicles and organelles in the cell, resulting in diverse exosome compositions [5, 6]. Microvesicles are vesicles 100–1000 nm in diameter formed by the budding of the plasma membrane. Apoptotic bodies are 100–5000 nm in diameter, making them larger than exosomes, and originate from apoptotic cells containing genomic DNA. Different EV subtypes cannot be completely categorized according to size or density because EVs within the exosome size range have the same biophysical properties [7] (Fig. 1).

## Separation and purification of extracellular vesicles

The specific method to separate and purify EVs is crucial for successful isolation. Isolation and purification of exosomes are key to understanding their mechanism and potential function. The main methods for separating exosomes are differential ultracentrifugation, ultrafiltration, size-exclusion chromatography, immunoaffinity capture, and microfluidic techniques [8]. Differential centrifugation, a traditional method for separating various mixtures of EVs, is unsuitable for isolating exosomes from clinical samples because it is time-consuming, laborious, and requires expensive equipment [9]. Ultrafiltration and size-exclusion chromatography have been used to prepare high-purity exosomes. Ultrafiltration is based on size separation, which is fast and does not require expensive equipment. However, its drawback is the difficulty in removing contaminating proteins [10]. Size-exclusion chromatography can separate exosomes from proteins but not liposomes, macromolecules, or particles, and the equipment is expensive. Immunoaffinity capture is used to selectively capture specific exosomes from complex samples, which is fast, convenient, and less demanding. To improve the isolation of EVs, most researchers use one or more additional techniques after the first step, such as ultrafiltration after differential centrifugation, application of a density gradient, or chromatography [11, 12]. Various technologies or combinations are currently being developed, and if they yield higher efficiencies than traditional methods, some of them may become more prominent in subsequent years.

Traditional methods have many drawbacks, such as low yield and purity, high equipment cost, and time-consuming processes. Therefore, there are still many challenges in the clinical isolation of high-purity exosomes. Recently, microfluidic technology has become an important technique for separation, detection, and analysis based on the biophysical properties of exosomes. This technique employs usual separation determinants while implementing new separation mechanisms, such as acoustic, electrophoresis, and electromagnetic operations, which are fast and use fewer samples and reagents, rendering EV separation more efficient and convenient [13, 14].

## Identification of extracellular vesicles

The study of the physicochemical properties of exosomes plays an important role in understanding their biological interactions. There are many methods to study the characteristics of exosomes, such as transmission electron microscopy (TEM), RNA sequencing, nanoparticle tracking analysis (NTA), western blotting, flow cytometry, and resistance pulses [15, 16]. TEM is a commonly used technique for the characterization of exosomes, which are often saucer-like and have a lipid bilayer [17]. In addition, exosomes can be characterized by the presence of RNA, whose content can be determined by global RNA profiling. NTA tracks each particle using image analysis to measure the concentration, movement, and size distribution of exosomes. This method allows rapid sample preparation, preserving the original form of the sample after measurement. Western blotting is a commonly used and recognized method for identifying intravesicular or membrane protein markers. The types of markers include three yang and one yin: supra-membrane, intra-membrane, cell source, and no organelle. In particular, the markers typically found on the membrane of exosomes are transmembrane proteins, including CD63, CD9, CD81, and CD82, suggesting that exosomes have a cell-derived membranous structure. The intra-membrane markers Alix and Tsg101 indicate that exosomes have an intact membrane structure [18], and the tissue-specific markers CD90 (MSC) and CD9 (MSC, T, NK) identify the type of cell from which exosomes originated. Flow cytometry, a suitable technique for reproducible analysis of clinical samples, allows the analysis of different physicochemical properties of cells and particles in suspension, enabling size and structure assessment of exosomes [19, 20]. Resistance pulse sensing can be used to measure the size distribution and concentration of exosomes. The inherent heterogeneity of exosomes prompts us to combine quantitative techniques to distinguish them, which might help develop new methods to isolate and identify exosomes.

## Tracing extracellular vesicles

The in vitro labeling or in vivo tracing of separated exosomes is beneficial for further investigating the functions of exosomes. The latest tracing methods include labeling exosomes with fluorescent membrane dyes, for instance, PKH67 (green fluorescence), PKH26 (red fluorescence), DIO (red fluorescence), and FM4-64 (red fluorescence). PKH67 and PKH26 can bind stably to the lipid region of the cell membrane and emit fluorescence. However, its limitation lies in the formation of dye aggregates in an aqueous solution similar to exosomes, modifying the structure of exosomes and changing their functions and physical properties [21]. DIO, one of the most commonly used cell membrane fluorescent probes, can only emit strong green fluorescence when it enters the cell membrane. FM4-64 dye is a lipophilic and water-soluble styrene compound that can specifically combine with the plasma membrane and intimal organelles to emit red fluorescence. SYTORNA Select can be combined with RNA to emit green fluorescence, which can be used to label RNA in exosomes. The exosomes membrane is rich in three transmembrane protein families (CD63, CD81, and CD9) and three heat shock protein families (HSP60, HSP70, and HSP90) involved in exosome transport. By coupling CD63 or CD81 with fluorescent proteins, fluorescent membrane proteins are expressed on the exosome membrane [22, 23].

Aside from the fluorescence imaging tracer method, Betzer et al. [24] tracked exosomes based on glucose-coated gold nanoparticle (GNP) labeling combined with CT imaging. They found that the GNPs were transported by glucose transporter (GLUT-1) and involved endocytosis activity. The authors further confirmed that this technique can be used as a diagnostic tool for various brain diseases and may enhance exosome-based neuronal recovery therapy. However, this technique needs to label exosomes directly, and it is impossible to determine whether the signal in the image refers to the actual exosome or the leaked or transferred content; furthermore, this technique is tainted by issues related to ionizing radiations. Jung et al. [25] labeled exosomes with superparamagnetic iron oxide nanoparticles (SPIONs) and performed longitudinal imaging by MRI. Results showed that the image after intravenous injection could penetrate the deep structure with high resolution and to the maximum degree. However, during the whole process, the nanoparticles could not be completely included in the exosome, and the resulting image may have been produced by the fusion of SPION exosomes into the cell. Therefore, magnetic imaging may not necessarily provide information about the ultimate fate of exosomes. In addition, nuclear imaging using radioactive materials is widely used for cell imaging, and radiation emitted by radionuclides can be detected in the body with a special camera. Nuclear imaging can help visualize deep structures and organs by improving sensitivity and tissue penetration. However, because radionuclides have short half-lives, they offer relatively short imaging and tracking period.

Photoacoustic imaging (PAI) is a recently developed hybrid biomedical imaging method based on the photoacoustic effect that combines ultrasonic and optical imaging. PAI is an attractive non-invasive method that couples the advantages of high spatial resolution and deep penetration of ultrasonic imaging with the high contrast of optical imaging. In addition, bioluminescence imaging technology can provide whole-body images of biological distribution, and because the markers are inherent, exosome tracking is highly reliable. However, the substrate has a short half-life and must be injected before each imaging session. Therefore, a fast and accurate imaging system that can be used over long periods is needed. In addition, any fluorescence-based in vivo imaging is limited in resolution and penetration depth and cannot clearly show the position of exosomes in the deep body structure, so this technique is the most suitable for preclinical research. In conclusion, for tracing exosomes, we need to select the most appropriate tracer method for the experimental purpose.

## Contents and modes of action

EVs are intercellular transport systems with multiple functions in the human body. An exosome is an EV produced by various cells, which is rich in cell-derived proteins, DNA, RNA, lipids, and metabolites and can be transferred from parent cells to other cells. Larger microbubbles are closer to cells in composition. Although the physiological purpose of exosome production needs to be studied to a large extent, evidence suggests that they are agents of cell-to-cell communication in health and disease, affecting all aspects of cell biology. It was found that the size, content, and function of exosomes from different cell sources are highly heterogeneous [26–28]. The uneven size of exosomes, the microenvironment, and the inherent biological behavior of cells will affect the content of exosomes and their biomarkers.

To determine the therapeutic potential of EVs, a well-designed in vivo application is essential. To correctly measure the therapeutic effect of the cargoes carried by EVs, an accurate and reliable drug administration strategy is important. Recently, Gupta et al. [29] reviewed the current preclinical literature and clinical use of EVs/exosomes doses, classifying and summarizing them according to different separation methods (purification or not), measurement methods (protein quantity/particle number), experimental animals (rats, mice, or pigs), administration routes (systemic/local), research areas (cardiovascular, neurological, inflammation, or cancer), and clinical trials. Finally, the authors concluded that, despite differences in methods and doses in different study types (in vitro or in vivo) and models (different cells or animals), EVs could potentially benefit the treatment of various diseases. Although there has been a lack of consistency between studies attempting to clinically apply EVs, their potential clinical application has opened up an exciting new way to provide effective treatment. With increasing interest in this field, unified detection methods and standard operating procedures must be established to reduce inconsistencies between studies.

## Biological function

Each exosome has many surface molecules that can interact with a series of homologous ligands on the plasma membrane and endocytosis membrane of the receptor cell, thus entering the receptor cell. Exosomes are continuously produced by donor cells and absorbed by recipient cells, but whether production and absorption are carried out synchronously or not remains to be elucidated [30, 31]. After exosomes are produced by donor cells, they are present in various body fluids and are rich in macromolecules. However, in some cases, exosomes exclusively uptake nutrients. In addition, exosomes play vital roles in different biological processes, such as transmitting signals and functional macromolecules to recipient cells, mediating intercellular signal transduction, and participating in physiological processes such as angiogenesis, inflammation, and apoptosis. Exosomes derived from allogeneic mesenchymal stem cells (MSCs) carry multiple miRNAs and regulate cell cycle progression, proliferation, and angiogenesis, which involves multiple physiological processes [32–34]. Exosomes are involved in differentiation, neovascularization, blood flow recovery, and capillary network formation. Exosome-mediated inflammatory responses are involved in tumor development, immune monitoring, and treatment resistance. The study of the relationship between inflammation and exosome molecular levels is a key step in understanding possible biomarkers of inflammatory diseases. Apoptotic bodies are a class of EVs released by dead cells as products of apoptotic cell decomposition [35, 36]. Therefore, exosomes can be used to reveal the mechanism of disease development, for early diagnosis and disease assessment, and can also be used as a drug delivery system to treat diseases. Exosomes also play an important role in different types of cancer, neurodegenerative and infectious diseases, pregnancy complications, autoimmune diseases, and other pathological conditions [37, 38].

## Research progress on extracellular vesicles in the pathogenesis of pSS

PSS is a multi-system autoimmune disease characterized by a dry mouth, ocular dryness as the main feature, lymphocytic infiltration, and the presence of multiple autoantibodies. Exosomes mediate intercellular signaling and participate in multiple physiopathological processes, playing an important role in different biological processes. Studies have shown that biomolecules in exosomes can reflect specific pathological conditions and are therefore considered potential biomarkers. Nevertheless, although the internationally recognized diagnostic criteria of SS include oral and eye symptoms, objective glandular secretion, and laboratory abnormalities, atypical clinical manifestations and laboratory-negative SS patients have been documented; thus, we need more sensitive biomarkers to enable an early diagnosis.

In 2005, Kapsogorgou et al. [39] showed that exosomes produced from salivary gland epithelial cells (SGECs) of pSS patients contain a large number of cytokeratins, in addition to previously identified protein types, such as actin or tubulin, which are epithelium-specific markers. Kapsogorgou et al. further demonstrated for the first time that intracellular autoantigenic Ro/SSA, LA/SSB, and Sm ribonucleoproteins are found in vesicles. Although secretion is not restricted to cells of pSS origin, it has also been suggested that exosomes may mediate the presentation of these autoantigens. Exosomes are thought to present antigens directly through their surface receptors or indirectly via loaded antigen-presenting cells (APC); however, the specific physiological significance in vivo warrants further investigation. Another study found that an EBV-specific microRNA (EBV-miR-BART13-3p) is highly expressed in salivary gland epithelial cells of pSS patients but not in healthy people and that this miRNA can be transferred from B cells to SGECs through exosomes, ultimately affecting salivary gland function [40]. Moreover, Cortes-Troncoso et al. [41] have shown that a specific miRNA, miR-142-3p, contained in T cell exosomes, can participate in Ca^2+^ signal transduction, cAMP production, and protein production in SGECs. Ca^2+^ and cAMP pathways are the main signal systems of secretory epithelium, which control the function of almost all secretory glands and are important for regulating the secretion of fluid and enzymes in exocrine glands. Activated T cells secrete miR142-3p-containing exosomes and transfer them to salivary gland cells, resulting in changes in the Ca^2+^ signaling pathway, limited cAMP production, and decreased protein production in salivary gland cells. Therefore, T cell-derived exosomes transfer miR-142-3p to SGECs, damaging the mechanism related to the secretory function of SGECs, resulting in dysfunctional SGECs in pSS patients, which is considered the pathogenic driver of pSS.

In the early stages of the disease, the patient’s symptoms are atypical, and serological tests are negative. Therefore, a more accurate diagnostic method is needed [42, 43]. Michael et al. [44] isolated exosomes from the saliva of the parotid gland of patients with pSS for the first time, and these exosomes contained a sufficient amount of miRNA. Exosomes isolated from the salivary gland come from specific cells in the gland, which can indicate the physiological state of the gland at the protein and regulatory levels. Thus, the miRNAs contained in the salivary exocrine not only participate in the physiological and pathological processes of various salivary gland diseases but can also be used as a biomarker. It is necessary to investigate further the value of exosomes and exosome miRNAs in the diagnosis and prognosis of salivary gland diseases. Similarly, EVs isolated from stimulated saliva and tears of 27 patients with pSS and 32 healthy controls were analyzed by liquid chromatography–mass spectrometry (LC–MS), and a biomarker map of EV proteins was established. The expression of innate immune-related proteins, cellular signal transduction proteins, and wound repair-related proteins was found in salivary EVs of pSS patients. Saliva EVs are vital biomarkers for activating the immune system and adipocyte differentiation. Tear EVs showed overexpression of tumor necrosis factor-α signal transduction-related proteins and B cell survival-related proteins. LC–MS analysis of saliva and tear EVs of pSS patients can screen new biomarkers that may contain saliva and lacrimal gland disease targets, which can improve the diagnostic accuracy of pSS and may also be used for disease staging and monitoring [45, 46]. Recently, Kakan et al. [47] used small RNA deep sequencing to identify the characteristics of serum exosome miRNA in early and middle autoimmune lacrimal gland inflammation and pSS mouse models. They found seven miRNAs with the most significant differences in expression. miRNAs are the main regulators of gene expression, and due to the complex causes of pSS, this group of significantly differentially expressed miRNAs may become a new biomarker for diagnosing pSS. Despite these advances, there is not enough evidence to show that exosomes or exosomal miRNAs can be used as reliable markers of pSS. In addition, there has been no study on the use of exosomes to evaluate the activity and prognosis of pSS disease [48–50]. Therefore, further studies are needed to confirm the potential role of exosome or exosome miRNAs as specific biomarkers of Pss [51, 52].

Until now, studies on exosomes of pSS have mainly focused on saliva and tears [53]. However, pSS is a multi-system disease, and exosomes of other tissues and organs still need to be investigated. Sellam et al. [54] found that autoimmune diseases are characterized by cell activation, and a common feature of activated cells is that they can shed fragments from the plasma membrane. These fragments represent heterogeneous populations of membrane-covered vesicles with a diameter of 0.1–1 μm called microparticles (MPs). The results showed that the level of circulating MPs increased in pSS, SLE, and RA could be used as a biomarker of systemic cell activation. Besides, the level of MPs derived from leukocytes increased only in pSS, while in severe cases, the level of MPs was lower, possibly due to the high activity of secretory phospholipase A2 (SPLA2), which leads to the consumption of MPs. In patients with pSS, platelet-derived MPs, sCD40L, and sCD62P increased, highlighting platelet activation. Subsequently, studying 34 patients with pSS and 18 healthy controls, Bartoloni et al. [55] found that circulating endothelial microparticles (EMPs) and endothelial progenitor cells (EPCs) in patients with pSS can be used as markers of endothelial injury. The increased proportion of EMP/EPC can destroy the integrity of the endothelial wall, and increase the risk of thrombosis, leading to endothelial dysfunction, a cardiovascular risk factor.

## Research progress on extracellular vesicles in the treatment of pSS

PSS is a multi-system autoimmune disease characterized by lymphocyte infiltration and a variety of autoantibodies in the salivary and lacrimal glands, leading to a dry mouth and dry eyes. Currently, there is no effective treatment for pSS because pSS treatment is complex [56, 57]. Although saliva substitutes and artificial tears can relieve symptoms, NSAIDs, glucocorticoids, and immunosuppressants such as methotrexate, mycophenolate mofetil, and biological agents can alleviate immune inflammation in advanced patients [58], the long-term use of these drugs can cause side effects. Exosomes have been extensively studied because of their important physiological and pathological roles in autoimmune diseases. Firstly, exosomes can be separated from various body fluids and are stable after long-term preservation; secondly, exosomes have a relatively long half-life in vivo; thirdly, exosomes can wrap bioactive substances and protect them from being degraded by enzymes; lastly, exosomes can be further modified to meet the needs of specific treatment schemes.

In the past few decades, stem cell therapy has been widely used to treat diseases and injuries, and research on MSCs is the most extensive [59–61]. Stem cell therapy offers a wide range of immunomodulatory and tissue repair capabilities and is considered a new strategy for treating autoimmune and inflammatory diseases [62]. It has been found that intravenous infusion of bone marrow- or the umbilical cord-derived MSCs can alleviate the symptoms of pSS [63]. In a murine model of pSS, allogeneic MSCs transplantation improved salivary gland secretion, reduced salivary gland lymphocyte infiltration, and facilitated tear secretion. When the salivary gland is injured, MSCs can differentiate into SGECs, acinar cells, and vascular endothelial cells because of the potential for multiple differentiation, which can repair the gland and restore the function of the gland [64]. However, MSCs therapy also has some limitations, such as limited expandability of MSCs, great heterogeneity in biological characteristics caused by donors and different amplification methods, lack of standard methods to detect the therapeutic effect of these cells, and difficulties in applying MSCs autologous transplantation because of the long preparation period and short opportunity for cell transplantation [65].

Recently, it has been established that EVs carry bioactive molecules in the same way as their progenitor cells and can overcome the difficulties of MSCs therapy. Mesenchymal stem cell-derived exosomes (MSCs-exos) can simulate the immune regulation and tissue repair of MSCs. MSCs-exos offer a wide range of sources, more stable properties, no heterologous risk, no functional attenuation with time, and stronger induction signals. They can be used as nanocarriers for nucleic acids, proteins, and small molecular drugs. Hai et al. [66] tried to differentiate MSCs from induced pluripotent stem cells (iPSCs), and iPSC-MSCs inhibited the infiltration of salivary gland lymphocytes in the NOD mouse pSS model in the same way as BM-MSCs. iPSC-MSCs and their derived EVs are expected to prevent pSS progression before the onset of sialitis through a mechanism related to the inhibition of Tfh and Th17 cell differentiation and APC activation. Subsequently, Abughanam et al. [67] found that MSCs extract (MSCsE) can also relieve the dry mouth and dry keratoconjunctivitis in pSS-like diseases. MSCsE promotes the expression of key genes in salivary and lacrimal gland functions, repair, and regeneration.

Previous studies have shown that bone marrow-derived suppressor cells (MDSCs), the precursors of bone marrow-derived DCs, macrophages, and/or granulocytes, show impaired immunosuppressive function during the disease in experimental Sjögren’s syndrome (ESS) mice. However, whether maintaining or restoring the inhibitory function of MDSCs has therapeutic significance for pSS remains to be determined. Recently, Rui et al. [68] found that exosomes derived from mouse extra-olfactory MSCs (OE-MSCs-Exo) can significantly enhance the inhibitory function of MDSCs by upregulating the expression of arginase and increasing ROS and NO levels. Simultaneously, intravenous injection of OE-MSCs-Exos significantly delayed disease progression and restored MDSC function in ESS mice. IL-6 released by OE-MSCs-Exos activates the MDSCJAK2/STAT3 pathway. In addition, S100A4 in OE-MSCs-Exos mediates autocrine IL-6 signaling through the TLR4 signaling pathway, suggesting that OE-MSCs-Exos delay the progression of ESS by enhancing the immunosuppressive function of MDSCs, which may constitute a new strategy for the treatment of pSS and other autoimmune diseases.

Moreover, Li et al. [69] found that treatment with labial MSCs (LGMSCs) and their exosomes (LGMSCs-Exos) reduced the inflammatory infiltration of salivary glands and restored the secretory function of salivary glands in NOD mice. It was also found that LGMSCs or LGMSCs-Exos inhibited Th17 cell differentiation in vitro but promoted the induction of Treg cells from peripheral blood mononuclear cells of NOD mice and pSS patients, accompanied by a decrease in the levels of IL-17, interferon-γ, and IL-6, and an increase in T cell secretion of transforming growth factor-β and IL-10. At the same time, in TLR4-stimulated splenocytes and a mouse model of pSS, Kim et al. [70] found that the EVs of iPSCs in the early passage had a better immunomodulatory effect than the EVs of iPSCs in the late passage. It was found that TGF-β1, TGF-β2, and miR-21 were enriched in early passage iPSCs EVs, while miR-125b was enriched in late passage iPSCs EVs. Importantly, in the early passage of bone marrow-derived MSCs, the regulation of TGF-β1, miR-21, and miR-125b significantly changed the inhibitory effect of their EVs on the production of Th1 and Th17 type cytokines. Furthermore, in the late passage of iPSCs, miR-21 overexpression and miR-125b inhibition improved the regulatory function of EVs. Therefore, TGF-β1 and miR-21 are the key factors mediating EV-mediated immune regulation, while miR-125b is a negative regulator. The development of exosome-based therapies faces many challenges, especially the production of extracellular preparations, which is a major hurdle to therapeutic applications because of their heterogeneity and low productivity. In addition, it plays an important role in improving the therapeutic potential and delivery efficiency of exosomes; thus, large-scale clinical application is still in its infancy [71].

Exosomes participate in a wide range of biological processes in cells. There is evidence that engineered exosomes containing therapeutic agents can reduce the carcinogenic activity of human cancer cells. Exosomes are a promising and efficient drug delivery system in the medical field, with broad application prospects in clinical trials, which may also be used to treat pSS [72–74]. In addition, exosomes have become a promising drug delivery system to target specific areas because of their safety, low immunogenicity, high compatibility, and long half-lives. Some chemicals, proteins, and antibodies have been modified on the surface of exosomes to build a targeted and multifunctional biological drug delivery system for enhanced therapy [75, 76]. The research scenario on EVs in pSS is summarized in Table 1.

**Table 1:** Research of EVs in pSS

Patients/animals	EVs	Source	Content	Function	References
Patients	Exos	SGECs	Ro/SSA, La/SSB, and Sm RNPs, epithelial-specific cytokeratins	Exos are thought to present antigens directly through their surface receptors or indirectly through antigen-loaded APCs	[39]
Patients	Exos	B cells	EBV-miR-BART13-3p	EBV-miR-BART13-3p can be transferred from B cells to SGECs to affect the function of salivary glands	[40]
Patients	Exos	T cells	miR-142-3p	miR-142-3p contained in T cell-derived Exos can participate in Ca^2+^ signal transduction, cAMP production, and protein production in SGECs, affecting the function of salivary glands	[41]
Patients	Exos	saliva	miRNA	miRNAs contained in salivary exocrine can be used as biomarkers and may play an important role in the diagnosis and prognosis of various salivary gland diseases	[44]
Patients	EVs	Saliva tear	Proteins	EV proteomic analysis is used to screen potential biomarkers in saliva and tears of pSS patients to improve the diagnostic accuracy and may also be used for disease staging and monitoring	[45]
Patients	MPs	Plasma	–	The level of plasma MPs in patients with pSS is increased, which can be used as a biomarker to reflect cell activation	[54]
Patients	MPs	Circulating	–	EMP and EPC can be used as markers of endothelial injury in patients with pSS	[55]
NOD mice	Exos	Serum	miRNA	The sequencing of miRNA in serum Exos identified seven miRNAs that were upregulated in NOD mice, which may become new biomarkers for the diagnosis of pSS	[47]
NOD mice	EVs	iPSCs	–	EVs are expected to prevent the progression of pSS before the onset of sialitis through a mechanism related to the inhibition of Tfh and Th17 cell differentiation and APC activation	[66]
NOD mice	MSCsE	MSCs	MSCsE	MSCsE alleviates dry mouth and keratoconjunctivitis in pSS-like diseases and promotes the expression of key genes involved in salivary and lacrimal gland function, repair, and regeneration	[67]
C57BL/6 mice	Exos	OE-MSC	–	Exos derived from mouse extra-olfactory MSCs can significantly enhance the inhibitory function of MDSCs by upregulating arginase expression and increasing the levels of ROS and NO	[68]
NOD mice/PBMC	Exos	LGMSCs	–	LGMSC-Exos reduced the inflammatory infiltration of salivary glands in NOD mice, restored the secretory function of salivary glands, inhibited the differentiation of Th17 cells in vitro, promoted the induction of Treg cells in PBMC of NOD mice and pSS patients	[69]
TLR4-stimulated splenocytes and pSS mice	EVs	iPSCs	–	TGF-β1 and miR-21 are key factors mediating EV-mediated immune regulation, while miR-125b is a negative regulator	[70]

## Prospect of clinical transformation of EVs in pSS

For many years, researchers have been trying to develop better diagnosis and treatment methods for patients with pSS. Increasing evidence shows that exosomes can be used as biomarkers and therapeutic tools for pSS, and we summarized the role of exosomes in pSS and their potential as biomarkers [77]. In addition to acting as biomarkers, EVs are considered a potential therapy, which can be divided into four categories: (1) EVs to transfer the contents of EVs to induce immunosuppressive or immunostimulatory effects, including antibacterial, anti-inflammatory, and anti-tumor effects, (2) EVs as an alternative to MSC transplantation, (3) EVs as a new vaccine for the treatment of tumors or infections, and (4) exosomes as natural nano-biological carriers that are stable, have permeable membranes, and can pass through the blood–brain barrier. Transporting therapeutic goods through exosomes is more efficacious and leads to fewer missing effects than using other biological carriers (such as liposomes) because exosomes can “recognize” specific cells. Therefore, exosomes have become a tool for the delivery and transfer of drugs, miRNAs, small interfering RNAs, and other compounds that remain stable in exosomes to treat cancer and other diseases [78, 79]. Extensive research on EVs is essential before applying EVs to the clinical diagnosis, prognosis, and treatment of autoimmune diseases. Future research should include (1) isolation and purification of EVs, (2) in-depth understanding of the biogenesis and targeting of EVs, (3) an in-depth understanding of the mechanism of immunostimulation and immunosuppression induced by EVs, (4) evaluation of the efficacy and reliability of EVs as a nano-preparation or in vivo drug delivery system, and (5) clinical application.

## Conclusion

In this review, we summarized the isolation, purification, identification, tracing, potential as biomarkers, and therapeutic merit of exosomes in pSS. However, there is still a gap in understanding the biogenesis and role of exosomes, and it is not completely clear whether exosomes can be used as biomarkers and treatments in pSS and other autoimmune diseases. Currently, methods to isolate, purify, identify, and track exosomes and exosome-contained miRNAs are tedious and complicated. However, a comprehensive study of exosomes is quintessential to implementing them in the diagnosis and treatment of SS and other autoimmune diseases. Exosomes can be traced more accurately to assess whether they reach target cells, causing a series of reactions. In conclusion, although the clinical application of exosomes to pSS is still in its early stages, further research might help develop more advanced, exosome-based treatments.

## Data Availability

Please contact the corresponding author for data requests.

## Abbreviations

pSS: Sjögren’s syndrome
NSAIDs: Non-steroidal anti-inflammatory drugs
EVs: Extracellular vesicles
TEM: Transmission electron microscopy
NTA: Nanoparticle tracking analysis
GNP: Glucose-coated gold nanoparticle
GLUT-1: Glucose transporter
SPIONs: Superparamagnetic iron oxide nanoparticles
PAI: Photoacoustic imaging
MSCs: Mesenchymal stem cells
SGECs: Salivary gland epithelial cells
APC: Antigen-presenting cells
LC–MS: Liquid chromatography–mass spectrometry
MPs: Microparticles
SPLA2: Secretory phospholipase A2
EMPs: Endothelial microparticles
EPCs: Endothelial progenitor cells
Exos: Exosomes
MSC-exos: Mesenchymal stem cell-derived exosomes
IPSCs: Induced pluripotent stem cells
MSCsE: MSCs extract
NOD: Non-obese diabetic
OE-MSCs: Olfactory ecto-mesenchymal stem cell
MDSCs: Marrow-derived suppressor cells
ESS: Experimental Sjögren’s syndrome
LGMSCs: Labial mesenchymal stem cells
BM: Bone marrow
iMSCs: Induced pluripotent stem cells
PBMC: Peripheral blood mononuclear cells
Treg: Regulatory T cells
Th17: T helper 17 cells
